# Postoperative early ambulation in neurosurgical patients: influencing factors and nursing strategies

**DOI:** 10.3389/fneur.2025.1666297

**Published:** 2025-11-19

**Authors:** Yan Xu, Xiangyi Yin, Yi Xia, Xing Qian

**Affiliations:** 1 Department of Nursing, Suzhou Hospital, Affiliated Hospital of Medical School, Nanjing University, Suzhou, China; 2 Department of Neurosurgery, Suzhou Hospital, Affiliated Hospital of Medical School, Nanjing University, Suzhou, China; 3 Department of Anesthesiology, Suzhou Hospital, Affiliated Hospital of Medical School, Nanjing University, Suzhou, China

**Keywords:** postoperative early ambulation, neurosurgical patients, influencing factors, nursing intervention, clinical practice

## Abstract

**Background:**

Early postoperative ambulation holds significant implications for patient prognosis. The present study aims to analyze the implementation rate of early postoperative ambulation in neurosurgical patients, identify its independent influencing factors, and provide evidence-based support for optimizing clinical treatment and nursing interventions.

**Methods:**

The study population comprised neurosurgical patients admitted to a tertiary medical center in Jiangsu Province, China, between January 2024 and May 2025. Comprehensive perioperative data—encompassing baseline demographics and early postoperative ambulation status—were analyzed to identify influencing factors of early postoperative ambulation.

**Results:**

A total of 268 neurosurgical patients were included, among whom 85 (31.7%) achieved early postoperative ambulation. Correlation analyses demonstrated that age (*r* = −0.586), body mass index (BMI; *r* = −0.520), presence of a postoperative drainage catheter (*r* = −0.590), duration of surgery (*r* = −0.587), and postoperative Visual Analog Scale (VAS) score (*r* = −0.614) all exhibited significant inverse associations with early postoperative ambulation. Logistic regression analysis identified five independent factors influencing early postoperative ambulation: age ≥ 60 years (odds ratio [OR] = 2.859, 95% confidence interval [95%CI]: 1.532–3.654), BMI ≥ 24 (OR = 2.194, 95%CI: 1.126–3.047), presence of a postoperative drainage catheter (OR = 2.706, 95%CI: 1.830–3.625), surgical duration ≥ 120 min (OR = 1.973, 95%CI: 1.285–2.585), and postoperative VAS score ≥ 3 (OR = 3.142, 95%CI: 2.869–3.704).

**Conclusion:**

The rate of early postoperative ambulation completion among neurosurgical patients is relatively low, with multiple factors influencing this outcome. Targeted nursing interventions are warranted to improve the implementation of postoperative ambulation in neurosurgical patients.

## Introduction

Neurosurgical procedures that directly target central nervous system lesions inevitably trigger substantial surgical trauma and functional risk. Intraoperative retraction, electrocautery, and tissue resection can disrupt neuronal networks and their projections, precipitating deficits in language, motor, or sensory domains (1). These focal injuries are further amplified by systemic responses: activation of inflammatory cascades, the hypothalamic–pituitary–adrenal axis, and the coagulation system collectively intensify postoperative recovery challenges (2, 3). Conventional practice has therefore favored strict bed rest to dampen intracranial pressure fluctuations and minimize the risks of wound bleeding or cerebrospinal fluid leakage. Yet prolonged immobilization introduces secondary harm: dependent atelectasis, venous stasis, and skeletal muscle disuse generate a cascade of complications—including hypostatic pneumonia, deep-vein thrombosis, and muscle atrophy—that prolong hospitalization and compromise long-term quality of life (4, 5).

Accumulating evidence from systematic reviews and randomized trials has redefined peri-operative care. Initiating graded ambulation within 24 h after surgery promotes recovery through several synergistic pathways: mechanical loading augments skeletal muscle perfusion and neurotrophin release, accelerating neuroplasticity and functional reorganization; positional changes reduce atelectasis, optimize ventilation–perfusion matching, and lower pulmonary infection rates; activation of the calf-muscle pump enhances venous return and decreases thrombotic risk; and restoration of gastrointestinal motility abbreviates postoperative ileus, improving nutrient utilization (6–8). Previous study (9) from patients with chronic subdural haematoma demonstrate that an early ambulation protocol commencing within 6 h post-surgery reduces complications without increasing recurrence or hemorrhage, underscoring the safety and efficacy of early ambulation in neurosurgical populations.

Nursing plays a pivotal role in facilitating early postoperative ambulation. Nurses are responsible for continuous monitoring of vital signs and neurological status during mobilization, pain assessment and management, patient education, and coordination with the multidisciplinary team. As highlighted by Shady et al. (10), standardized nursing interventions—such as individualized ambulation protocols, regular pain reassessment, and catheter care—are critical for improving patient outcomes after intracranial surgery. However, the integration of nursing-specific strategies into early ambulation programs remains understudied in neurosurgical settings, underscoring the need for targeted research in this area.

Despite these documented benefits, early postoperative ambulation remains inconsistently implemented in neurosurgical practice. The specific determinants—spanning patient characteristics and disease-related factors—have not been comprehensively elucidated. Therefore, the present study aims to identify and quantify the factors influencing early postoperative ambulation after neurosurgical procedures, thereby providing an evidence base for targeted therapeutic and nursing interventions in this high-risk cohort.

## Methods

### Study design and ethical consideration

This research adopted a retrospective cohort study design. This study has been approved by the ethical committee of Suzhou Hospital, Affiliated Hospital of Medical School, Nanjing University (approval number: IRB2025065). In addition, written informed consent was obtained from each enrolled patient prior to their participation in the study.

### Study population

We retrospectively screened consecutive neurosurgical patients treated at a tertiary academic hospital in Jiangsu Province, China, between January 2024 and May 2025. Inclusion criteria: (1) Index operation performed by the hospital's neurosurgical team; (2) Complete peri-operative data available in electronic medical records, nursing notes, and anesthesia charts; (3) Written informed consent provided by the patient or legal surrogate.

Exclusion criteria: (1) Transfer to another institution or discharge against medical advice within 72 h postoperatively; (2) >20% of key variables missing and unrecoverable.

### Definition of early postoperative ambulation

Early postoperative ambulation was defined as successful when, within 24 h of tracheal extubation (11, 12), all of the following conditions were met: (1) haemodynamic stability (systolic blood pressure 90–160 mmHg, heart rate 50–100 beats min^−1^, and either no vasoactive agents or a stable infusion rate for ≥2 h); (2) respiratory stability (SpO_2_ ≥ 92 % on room air or ≤ 2 L min^−1^ supplemental oxygen without continuous positive airway pressure); (3) a joint assessment by the attending neurosurgeon and the ward nurse confirming that the patient could follow commands; and (4) documentation in the nursing record of the patient standing and walking ≥5 m with the aid of a walker or staff assistance.

### Data collection

A total of 326 consecutive neurosurgical patients were treated at the tertiary academic hospital between January 2024 and May 2025; 268 were enrolled after applying inclusion and exclusion criteria. The following variables were extracted and collected from the institutional electronic health record: gender, age (years), body-mass index (BMI, kg m?^2^), documented history of hypertension, diabetes mellitus, and hyperlipidaemia, primary neurosurgical diagnosis, presence of a postoperative drainage catheter, and total operative time. Postoperative pain intensity was routinely quantified with the 100-mm visual analog scale (VAS) at rest within the first 24 h after extubation; scores were categorized as mild (1–3), moderate (4–6), or severe (7–10).

### Data analysis

All analyses were performed with SPSS 25.0 (IBM Corp., Armonk, NY). Categorical variables are reported as *n* (%), and continuous variables as mean ± SD. Between-group comparisons were conducted with the χ^2^ test for categorical data and the independent-samples *t*-test for normally distributed continuous data; non-normally distributed data were analyzed with the Mann–Whitney U-test. Variables associated with early postoperative ambulation were first screened by univariate analysis. Subsequently, factors with *P* < 0.10 in the univariate screen were entered into a multivariable logistic regression model to identify independent predictors of early postoperative ambulation. Correlations between early postoperative ambulation and individual characteristics were assessed with Pearson's r for normally distributed variables and Spearman's ρ for non-normally distributed variables. A two-tailed *P*-value < 0.05 was considered statistically significant.

## Results

Of the 268 enrolled neurosurgical patients, 85 (31.7 %) achieved early postoperative ambulation. As detailed in Table 1, the early postoperative ambulation and non-early postoperative ambulation groups differed significantly with respect to age, BMI, presence of a postoperative drainage catheter, duration of surgery, and postoperative VAS score (all *p* < 0.05). In contrast, gender distribution and the prevalence of hypertension, diabetes, hyperlipidaemia, or primary disease diagnosis did not differ between the two groups (all *p* > 0.05).

**Table 1:** The characteristics of included neurosurgical patients (*n* = 268).

**Variables**	**Early ambulation group (*n =* 85)**	**No early ambulation group(*n =* 183)**	***t*/χ^2^**	***p***
Male/female	51/34	106/77	1.942	0.103
Age(y)	56.28 ± 4.02	62.46 ± 5.84	1.870	0.041
BMI (kg/m^2^)	22.04 ± 2.16	24.90 ± 2.57	1.121	0.108
Hypertension	32(37.65%)	70(38.25%)	1.437	0.074
Diabetes	25(29.41%)	54(29.51%)	1.802	0.125
Hyperlipidemia	14(16.47%)	28(15.30%)	1.167	0.093
Disease diagnosis			1.588	0.060
Traumatic brain injury	36(42.35%)	80(43.71%)		
Brain tumor	21(24.71%)	42(22.95%)		
Cerebrovascular disease	15(17.65%)	32(17.49%)		
Other	13(15.29%)	29(15.85%)		
Presence of postoperative drainage catheter	16(18.82%)	96(52.46%)	1.626	0.016
Duration of surgery (min)	105.35 ± 42.08	146.87 ± 50.01	9.539	0.017
Postoperative VAS	2.856 ± 1.547	5.184 ± 1.933	1.763	0.021

As presented in Table 2, the correlation analyses indicated that age (*r* = −0.586), BMI (*r* = −0.520), presence of a postoperative drainage catheter (*r* = −0.590), duration of surgery (*r* = −0.587), and postoperative VAS score (*r* = −0.614) were significantly inversely associated with early postoperative ambulation (all *p* < 0.05).

**Table 2:** Correlation analysis of early ambulation and characteristics of included neurosurgical patients.

**Variables**	***r***	***p***
Gender	0.140	0.128
Age (y)	−0.586	0.031
BMI (kg/m^2^)	−0.520	0.048
Hypertension	0.129	0.105
Diabetes	0.102	0.118
Hyperlipidemia	0.144	0.127
Disease diagnosis	0.203	0.057
Presence of postoperative drainage catheter	−0.590	0.042
Duration of surgery (min)	−0.587	0.025
Postoperative VAS	−0.614	0.009

Table 3 outlines the variable assignments adopted for the multivariate logistic regression analysis conducted in this study. As depicted in Table 4, the logistic regression results identified five independent factors influencing early postoperative ambulation among neurosurgical patients (all *p* < 0.05). These factors included age ≥60 years (odds ratio [OR] = 2.859, 95% confidence interval [95%CI]: 1.532–3.654), BMI ≥ 24 (OR = 2.194, 95%CI: 1.126–3.047), presence of a postoperative drainage catheter (OR = 2.706, 95%CI: 1.830–3.625), surgical duration ≥ 120 min (OR = 1.973, 95%CI: 1.285–2.585), and postoperative VAS score ≥ 3 (OR = 3.142, 95%CI: 2.869–3.704).

**Table 3:** The variable assignment of multivariate logistic regression.

**Factors**	**Variables**	**Assignment**
Early ambulation	*Y*	Yes = 1, no=2
Age(y)	*X*_2_	≥60 = 1, < 60 = 2
BMI (kg/m^2^)	*X*_3_	≥24 = 1, < 24 = 2
Presence of postoperative drainage catheter	*X*_4_	Yes = 1, no = 2
Duration of surgery (min)	*X*_5_	≥120 = 1, < 120 = 2
Postoperative VAS	*X*_6_	≥3 = 1, < 3 = 2

**Table 4:** Logistic regression analysis on the influencing factors of early ambulation in neurosurgical patients.

**Factors**	**β**	***S* $\bar{x}$ **	**OR**	**95%CI**	***p***
Age ≥ 60 years	0.819	0.141	2.859	1.532–3.654	0.020
BMI ≥24	0.655	0.102	2.194	1.126–3.047	0.013
Presence of postoperative drainage catheter	0.829	0.266	2.706	1.830–3.625	0.019
Duration of surgery ≥120 min	0.644	0.183	1.973	1.285–2.585	0.036
Postoperative VAS ≥3	0.926	0.132	3.142	2.869–3.704	0.007

## Discussion

Only 31.7% of the 268 neurosurgical patients achieved early ambulation, a figure that is substantially lower than the 50–70 % reported after non-neurological surgery (13, 14). This discrepancy underlines the unique physiological burden of intracranial procedures—cerebral oedema, autonomic instability, and prolonged anesthetic wash-out—together with a culture of cautious bed rest aimed at preventing intracranial hypertension (15, 16). The low prevalence suggests that early ambulation should be adopted as an explicit quality-of-care metric for neurosurgical wards, analogous to enhanced recovery program in other specialties. Consistent with Shady et al. (10), our study emphasizes that nursing interventions—such as standardized pain management, catheter care, and individualized ambulation plans—are critical for improving early ambulation rates. Their framework for optimizing nursing care in intracranial surgery aligns with our proposed strategies, highlighting the need for nursing-led initiatives to address modifiable factors like pain and catheter-related immobility.

Age ≥ 60 years and BMI ≥24 kg m?^2^ independently doubled to tripled the odds of delayed ambulation. While chronological age is immutable, the associated sarcopenia and orthostatic intolerance can be mitigated. A 2-week pre-hospital “pre-rehabilitation” programme combining resistance training, protein supplementation, and orthostatic challenge has been shown to increase skeletal-muscle mass and reduce postoperative orthostatic hypotension in elderly oncological patients (17); a similar protocol merits pilot testing in neurosurgery. Obesity, conversely, is partially reversible. A 5 % preoperative weight loss, targeting visceral adiposity, improves respiratory mechanics and reduces the inflammatory milieu that delays mobilization (18, 19). In the immediate postoperative period, use of powered standing hoists and wide-base walkers may overcome the biomechanical disadvantage imposed by excess weight while ensuring patient safety (20).

Operations lasting ≥120 min and the retention of postoperative drainage catheters were robust predictors of immobility. Prolonged surgery reflects both case complexity and anesthetic depth; intraoperative use of processed EEG-guided anesthesia (e.g., BIS 40–60) reduces cumulative hypnotic dose and accelerates emergence, thereby shortening the time to first ambulation (21). Drainage catheters, although indispensable for hemorrhage surveillance, tether the patient to bed and amplify pain (22). A risk-stratified catheter protocol is proposed: low-risk cases (non-tumor, minimal brain manipulation) undergo early (< 24 h) removal after a negative CT scan, whereas high-risk cases receive a clamp-and-walk trial under continuous ICP monitoring (23). Adoption of smaller caliber, flexible drains and securement devices that permit greater range of motion may further reduce catheter-related immobility (24).

A VAS score ≥3 conferred the highest odds of delayed ambulation, yet neurosurgical patients often receive suboptimal analgesia due to concerns about over sedation and interference with pupillary monitoring. Severe pain can induce muscle guarding, limit range of motion, and reduce patient compliance with ambulation protocols (25). Neurosurgical procedures, which often involve sensitive neural structures, may be particularly prone to causing significant postoperative pain, making effective analgesia a critical component of enhanced recovery after surgery in this patient population (26, 27). Early physiotherapy sessions scheduled 30 min after analgesic administration exploit the window of maximal pain relief, improving compliance and reducing fall risk (28).

To translate the identified risk factors into measurable clinical gains, a coherent perioperative strategy must weave together geriatric pre-conditioning, catheter stewardship, operative efficiency, precision analgesia and real-time interprofessional feedback (29, 30). Elderly or overweight patients should undergo pre-operative screening for sarcopenia and orthostatic tolerance. A graded resistance and balance programme—beginning at the bedside and progressing to supervised ambulation within hours of extubation—should follow this screening. In addition to weight optimization, postoperative mobility for obese patients can be facilitated by powered standing hoists and wide-base walkers, which overcome biomechanical disadvantages while ensuring safety. Drainage catheters should be secured with low-profile devices and removed as soon as intracranial imaging and ICP dynamics permit, thereby eliminating the physical and psychological tether that discourages movement (31). Evidence-based checklists, pre-operative simulation and coordinated turnover enable surgical teams to compress operative time while preserving oncological and vascular safety. This in turn mitigates the cumulative inflammatory burden associated with delayed ambulation (32). Post-operative pain should be pre-emptively targeted with scalp infiltration of long-acting local anesthetics, scheduled multimodal agents and opioid-sparing adjuvants titrated to maintain VAS < 3, ensuring that analgesia enhances rather than impedes participation in physiotherapy. Continuous input from surgeons, anaesthesiologists, nurses and physiotherapists—facilitated by daily huddles and wearable accelerometry—allows dynamic recalibration of ambulation goals, transforming early ambulation from an aspiration into a reproducible neurosurgical quality metric (33, 34). Besides, patients with cardiovascular, pulmonary, or metabolic comorbidities require stratified mobilization strategies. For example, those with heart failure may benefit from gradual orthostatic progression (sitting → standing → walking) with continuous haemodynamic monitoring, while patients with chronic obstructive pulmonary disease may need supplemental oxygen during ambulation to maintain SpO_2_ ≥ 92%. Key factors for distinguishing suitable candidates for early ambulation include frailty indices (e.g., Clinical Frailty Scale), ASA physical status classification, and neurosurgical-specific risk scores (e.g., for hemorrhage or oedema risk). These metrics help identify patients for whom delayed mobilization may be safer, such as those with severe frailty (CFS ≥ 5) or ASA IV classification. It must be noted that multidisciplinary collaboration (surgeons, anaesthesiologists, nurses, physiotherapists, and geriatricians) is critical for these stratified decisions. Daily interprofessional huddles allow real-time assessment of comorbidity-related risks and adjustment of ambulation plans, ensuring safety and feasibility (35).

Several limitations warrant cautious interpretation of our findings. First, the single-center design and moderate sample size constrain external validity; practice patterns, referral criteria, and ethnic demographics specific to our tertiary neurosurgical institution may not be generalizable to other geographic regions or patient populations. Second, residual confounding remains plausible despite multivariable adjustment. Variables with potential implications for ambulation—such as preoperative frailty indices and postoperative delirium—were either incompletely documented or excluded from the analysis. Third, the observational nature of the study precludes definitive causal inference; unmeasured patient-level factors (e.g., psychosocial support, pain-coping mechanisms) and system-level variables (e.g., physiotherapist staffing ratios) may have introduced bias into the observed associations. Future multi-center prospective cohorts with larger, more heterogeneous populations are warranted to validate our risk model, which may enhance the translational potential of the proposed strategies in diverse clinical settings.

## Conclusion

In conclusion, the present study aimed to analyze early postoperative ambulation rates and their influencing factors in neurosurgical patients. Among 268 enrolled patients, only 31.7% achieved early ambulation, with age ≥60 years, BMI ≥24 kg/m^2^, surgical duration ≥120 min, postoperative drainage catheter retention, and VAS score ≥3 identified as independent influencing factors. These findings underscore the need for targeted interventions to improve early ambulation implementation in neurosurgical practice. These findings suggest that the early ambulation is determined by multiple factors. They include the interplay of baseline physiological reserve, procedural complexity, and nociceptive load. Given the complex factors influencing early ambulation, it is essential to explore corresponding effective interventions. Firstly, to counteract frailty-related deconditioning, we should strengthen the assessment of patients' physical conditions. Secondly, optimizing surgical efficiency can reduce the impact of surgical complexity on patients. Thirdly, we need to minimize catheter-associated immobility to promote patients' mobility. Moreover, titrating multimodal analgesia is necessary to relieve patients' pain and facilitate early ambulation.

In clinical practice, healthcare providers are advised to comprehensively evaluate patients' baseline physiological reserve before surgery. For instance, conduct detailed physical function tests and nutritional status assessments. Based on the evaluation results, customize early ambulation plans for different patients. In future research, it is recommended to expand the sample size, cover a wider range of patient types, and conduct long—term follow—up to observe the long—term impact of early ambulation on patient recovery. Explore more potential factors affecting early ambulation to improve the accuracy and comprehensiveness of the research.

## Data Availability

The raw data supporting the conclusions of this article will be made available by the authors, without undue reservation.
